# 4,7,13,18-Tetra­oxa-1,10-diazo­nia­bicyclo­[8.5.5]icosane hexa­fluorido­silicate

**DOI:** 10.1107/S1600536811026006

**Published:** 2011-07-09

**Authors:** Nalinava Sen Gupta, David S. Wragg, Mats Tilset, Jon Petter Omtvedt

**Affiliations:** a Centre for Accelerator Based Research and Energy Physics (SAFE), Department of Chemistry, University of Oslo, PO Box 1038 Blindern, Oslo 0318, Norway; b inGAP Centre for Research Based Innovation, Center for Materials Science and Nanotechnology, Department of Chemistry, University of Oslo, PO Box 1033 Blindern, Oslo 0315, Norway; c Department of Chemistry, University of Oslo, PO Box 1033 Blindern, Oslo 0315, Norway

## Abstract

The asymmetric unit of the title molecular salt, C_14_H_30_N_2_O_4_^2+^·SiF_6_^2−^, contains half of both the anion and the cation, both ions being completed by a crystallographic twofold axis passing through the Si atom. The cation has a cage structure with the ammonium H atoms pointing into the cage. These H atoms are shielded from inter­molecular inter­actions and form only intra­molecular contacts. There are short inter­molecular C—H⋯F inter­actions in the structure, but no conventional inter­molecular hydrogen bonds.

## Related literature

For related structures, see: Cos *et al.* (1982 ▶); Rehder & Wang (2003 ▶); Luger *et al.* (1991 ▶); Sen Gupta *et al.* (2011 ▶); Anderson *et al.* (2006 ▶); Braband *et al.* (2003 ▶); Llusar *et al.* (2001 ▶). For discussion of a cryptand as a mol­ecular automatic titrator, see: Alibrandi *et al.* (2009 ▶). For NMR data, see: Macchioni *et al.* (2001 ▶); Christe & Wilson (1990 ▶).
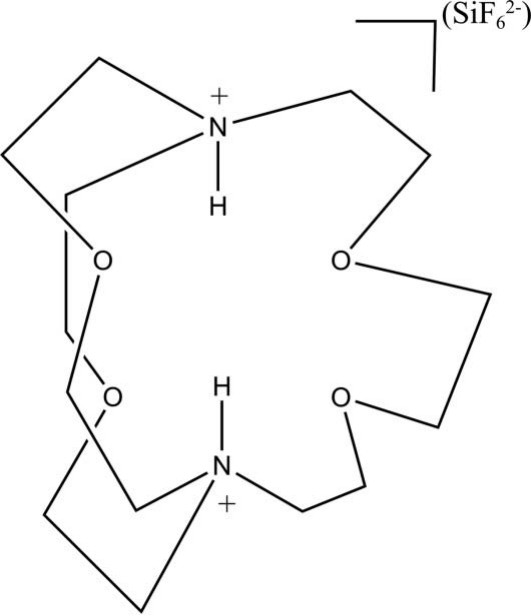


## Experimental

### 

#### Crystal data

C_14_H_30_N_2_O_4_^2+^·SiF_6_^2−^
*M* *_r_* = 432.49
Orthorhombic, 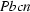

*a* = 10.050 (5) Å
*b* = 23.218 (5) Å
*c* = 8.031 (5) Å
*V* = 1874.0 (15) Å^3^
*Z* = 4
Mo *K*α radiation
μ = 0.21 mm^−1^
*T* = 293 K
0.11 × 0.10 × 0.05 mm

#### Data collection

Bruker SMART CCD area-detector diffractometer
Absorption correction: multi-scan (*SADABS*; Bruker, 2001 ▶) *T*_min_ = 0.977, *T*_max_ = 0.990
9809 measured reflections
2305 independent reflections
1467 reflections with *I* > 2σ(*I*)
*R*_int_ = 0.028

#### Refinement

*R*[*F* ^2^ > 2σ(*F* ^2^)] = 0.039
*wR*(*F* ^2^) = 0.116
*S* = 1.02
2305 reflections
123 parameters
H-atom parameters constrained
Δρ_max_ = 0.32 e Å^−3^
Δρ_min_ = −0.26 e Å^−3^

### 

Data collection: *SMART* (Bruker, 2001 ▶); cell refinement: *SAINT* (Bruker, 2001 ▶); data reduction: *SAINT*; program(s) used to solve structure: *SHELXS97* (Sheldrick, 2008 ▶); program(s) used to refine structure: *SHELXL97* (Sheldrick, 2008 ▶); molecular graphics: *DIAMOND* (Brandenburg & Berndt, 1999 ▶); software used to prepare material for publication: *publCIF* (Westrip, 2010 ▶).

## Supplementary Material

Crystal structure: contains datablock(s) I, global. DOI: 10.1107/S1600536811026006/fy2006sup1.cif

Structure factors: contains datablock(s) I. DOI: 10.1107/S1600536811026006/fy2006Isup2.hkl

Additional supplementary materials:  crystallographic information; 3D view; checkCIF report

## Figures and Tables

**Table 1:** Hydrogen-bond geometry (Å, °)

*D*—H⋯*A*	*D*—H	H⋯*A*	*D*⋯*A*	*D*—H⋯*A*
N1—H1⋯O2	0.91	2.19	2.701 (2)	115
N1—H1⋯O1	0.91	2.30	2.813 (2)	115
N1—H1⋯O1^i^	0.91	2.37	2.826 (2)	111
C1—H1*B*⋯F004^ii^	0.97	2.37	3.277 (3)	156
C011—H01*B*⋯F3^iii^	0.97	2.50	3.381 (3)	151
C2—H2*A*⋯F005^iv^	0.97	2.39	3.289 (3)	155
C014—H01*C*⋯F005	0.97	2.41	3.257 (3)	146
C3—H3*A*⋯F004^v^	0.97	2.41	3.189 (3)	137
C3—H3*B*⋯F3^iii^	0.97	2.17	3.129 (3)	169
C014—H01*D*⋯F3	0.97	2.50	3.039 (3)	115
C4—H4*B*⋯F005^v^	0.97	2.52	3.368 (3)	147

Symmetry codes: (i) 
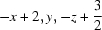
; (ii) 
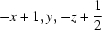
; (iii) 
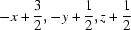
; (iv) 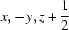; (v) 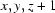.

**Fig. 1.**
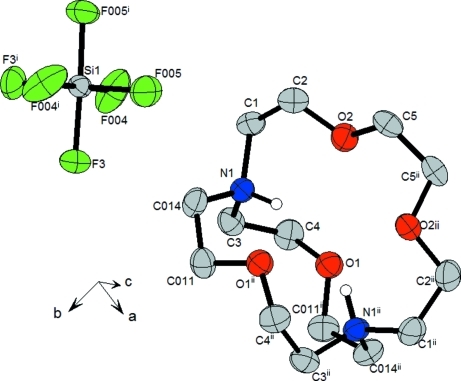
The molecular structure of (I), with atom labels and 50% probability displacement ellipsoids for non-H atoms. The hydrogen atoms attached to the carbons are omitted for clarity. Symmetry codes: (i) -x+1, y, -z+1/2; (ii) -x+2, y, -z+3/2.

**Fig. 2.**
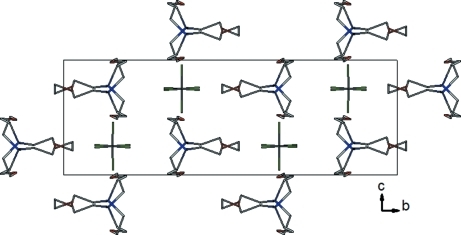
Packing diagram for (I) viewed along the a axis. H atoms omitted for clarity.

## supplementary crystallographic information

### Comment

Compound (I) was obtained unintentionally as the product of the attempted synthesis of a metal-encrypted tungsten(VI) complex with the [2.1.1]cryptand, 4,7,13,18-tetraoxa-1,10-diazabicyclo[8.5.5]icosane. We suspect that WCl_6_, being susceptable to hydrolysis, reacted with water that was present as a contaminant. Compound (I) was obtained by recrystallization of the crude reaction product from acetone. When the same product was recrystalized from methanol, a similar diprotonated cryptand salt with PF_6_^-^ as the anion formed (Sen Gupta *et al.*, 2011). The solvent used for recystallization was the only difference between the methods to obtain the two different crystals.

This structure was originally solved as a hexafluorophosphate salt, but the P—F bond lengths appeared unusually long and corresponded to typical Si—F rather than P—F bonds. The highest difference peak was close to H—N^+^, indicating that the occupancy of the H atom was higher than 0.5, which would be required to ensure charge neutrality in a PF_6_^-^ salt. The most negative difference density was observed near the central P atom, showing that in reality there are less electrons there. Furthermore, refinement of the data with Si gave slightly lower *R* factors than with P. Though similar long P—F bonds are not unprecedented (Braband *et al.*, 2003; Llusar *et al.*, 2001), the above observations very strongly suggested that this was a diprotonated SiF_6_^2-^ salt rather than a monoprotonated PF_6_^-^ salt.

The presence of both anions in the reaction product was confirmed by ^19^F NMR data collected in a CD_3_OD solution. The presence of the PF_6_^-^ anion was indicated by a doublet at δ = -74.7 p.p.m. with ^1^J(^19^F–^31^P) = 754 Hz (Macchioni *et al.*, 2001). A small singlet peak at δ = -130.6 p.p.m. and a heteronuclear coupling constant ^1^J(^29^Si–^19^F) = 109 Hz were also observed and correspond to the SiF_6_^2-^ anion (Christe & Wilson, 1990).

SiF_6_^2-^ is assumed to be generated by the formation of HF upon the hydrolysis of PF_6_^-^ and by the consequent reaction of HF with the silica of the glass. A smilar case was reported by Anderson *et al.* (2006).

In the crystal of compound (I), the two ammonium hydrogen atoms of the diprotonated cryptand cage are pointing inwards. Cryptands are known to form proton crypts, in which the protons are very efficiently concealed inside a tight molecular cavity. No exception is observed here: the ammonium hydrogen atoms are not involved in intermolecular hydrogen bonding. They only form intramolecular contacts with the oxygen atoms of the cryptand.

### Experimental

Reagents were purchased from Sigma-Aldrich and were used without further purification. Reactions were carried out under inert conditions by Schlenk-line techniques. The metal chloride (WCl_6_, 100 mg, 0.25 mmol) was allowed to stir for a minute in 10 ml toluene and then was reacted with a small excess of of AgPF_6_ (381 mg, 1.51 mmol) to give AgCl as a precipitate and W(PF_6_)_6_ dissolved in solution. After 30 minutes stirring, the precipitate was allowed to settle. The solution was transferred under inert conditions by cannula technique and treated with the solution of [2.1.1]cryptand (66 µl, 0.25 mmol) in 5 ml toluene for 30 minutes. The crude reaction product was obtained as dirty yellow mass after drying the solvent. Portions of the product were recrystallized from acetone which produced crystal (I).

### Refinement

Hydrogen *U*_iso_'s were set at 1.2 times the *U*_eq_ of the heavy atom to which the hydrogen was attached and refined in riding mode. C—H distances were fixed at 0.97 Å and the N—H distance at 0.91 Å.

### Crystal data

**Table:**

C_14_H_30_N_2_O_4_^2+^·SiF_6_^2^^−^	*F*(000) = 912
*M**_r_* = 432.49	*D*_x_ = 1.533 Mg m^−^^3^
Orthorhombic, *P**b**c**n*	Mo *K*α radiation, λ = 0.71073 Å
Hall symbol: -P 2n 2ab	Cell parameters from 2312 reflections
*a* = 10.050 (5) Å	θ = 2.2–28.0°
*b* = 23.218 (5) Å	µ = 0.21 mm^−^^1^
*c* = 8.031 (5) Å	*T* = 293 K
*V* = 1874.0 (15) Å^3^	Block, colourless
*Z* = 4	0.11 × 0.10 × 0.05 mm

### Data collection

**Table:**

Bruker SMART CCD area-detector diffractometer	2305 independent reflections
Radiation source: sealed tube	1467 reflections with *I* > 2σ(*I*)
graphite	*R*_int_ = 0.028
φ and ω scans	θ_max_ = 28.8°, θ_min_ = 2.2°
Absorption correction: multi-scan (*SADABS*; Bruker, 2001)	*h* = −11→13
*T*_min_ = 0.977, *T*_max_ = 0.990	*k* = −29→31
9809 measured reflections	*l* = −10→10

### Refinement

**Table:**

Refinement on *F*^2^	Primary atom site location: structure-invariant direct methods
Least-squares matrix: full	Secondary atom site location: difference Fourier map
*R*[*F*^2^ > 2σ(*F*^2^)] = 0.039	Hydrogen site location: inferred from neighbouring sites
*wR*(*F*^2^) = 0.116	H-atom parameters constrained
*S* = 1.02	*w* = 1/[σ^2^(*F*_o_^2^) + (0.0509*P*)^2^ + 0.6523*P*] where *P* = (*F*_o_^2^ + 2*F*_c_^2^)/3
2305 reflections	(Δ/σ)_max_ < 0.001
123 parameters	Δρ_max_ = 0.32 e Å^−^^3^
0 restraints	Δρ_min_ = −0.26 e Å^−^^3^

### Special details

**Table:**

Geometry. All s.u.'s (except the s.u. in the dihedral angle between two l.s. planes) are estimated using the full covariance matrix. The cell s.u.'s are taken into account individually in the estimation of s.u.'s in distances, angles and torsion angles; correlations between s.u.'s in cell parameters are only used when they are defined by crystal symmetry. An approximate (isotropic) treatment of cell s.u.'s is used for estimating s.u.'s involving l.s. planes.
Refinement. Refinement of *F*^2^ against ALL reflections. The weighted *R*-factor *wR* and goodness of fit *S* are based on *F*^2^, conventional *R*-factors *R* are based on *F*, with *F* set to zero for negative *F*^2^. The threshold expression of *F*^2^ > 2σ(*F*^2^) is used only for calculating *R*-factors(gt) *etc*. and is not relevant to the choice of reflections for refinement. *R*-factors based on *F*^2^ are statistically about twice as large as those based on *F*, and *R*- factors based on ALL data will be even larger.

### Fractional atomic coordinates and isotropic or equivalent isotropic displacement parameters (Å^2^)

**Table:**

	*x*	*y*	*z*	*U*_iso_*/*U*_eq_	
Si1	0.5000	0.14596 (3)	0.2500	0.02931 (19)	
F3	0.61848 (13)	0.19715 (5)	0.25800 (17)	0.0524 (3)	
O2	0.86157 (15)	0.02390 (6)	0.76498 (17)	0.0421 (4)	
F004	0.50972 (13)	0.14736 (7)	0.04208 (16)	0.0729 (5)	
F005	0.61920 (12)	0.09635 (5)	0.2602 (2)	0.0668 (4)	
O1	0.97909 (13)	0.14163 (5)	0.97904 (18)	0.0391 (3)	
N1	0.79018 (16)	0.13502 (6)	0.72125 (19)	0.0341 (4)	
H1	0.8702	0.1174	0.7367	0.041*	
C5	0.9322 (2)	−0.02623 (8)	0.7128 (3)	0.0403 (5)	
H5A	0.9389	−0.0267	0.5923	0.048*	
H5B	0.8844	−0.0605	0.7477	0.048*	
C1	0.6852 (2)	0.08871 (8)	0.7376 (3)	0.0450 (5)	
H1A	0.6581	0.0853	0.8531	0.054*	
H1B	0.6076	0.0991	0.6722	0.054*	
C3	0.7786 (2)	0.18006 (8)	0.8537 (3)	0.0394 (5)	
H3A	0.6854	0.1879	0.8757	0.047*	
H3B	0.8200	0.2155	0.8160	0.047*	
C011	0.9254 (2)	0.18663 (9)	0.5104 (2)	0.0412 (5)	
H01A	0.9253	0.2032	0.3996	0.049*	
H01B	0.9459	0.2168	0.5901	0.049*	
C4	0.8453 (2)	0.15967 (10)	1.0105 (3)	0.0429 (5)	
H4A	0.8456	0.1906	1.0917	0.051*	
H4B	0.7952	0.1278	1.0569	0.051*	
C2	0.7402 (2)	0.03213 (9)	0.6775 (3)	0.0483 (6)	
H2A	0.6785	0.0011	0.7013	0.058*	
H2B	0.7560	0.0333	0.5584	0.058*	
C014	0.7927 (2)	0.16036 (9)	0.5484 (2)	0.0424 (5)	
H01C	0.7736	0.1304	0.4677	0.051*	
H01D	0.7241	0.1896	0.5391	0.051*	

### Atomic displacement parameters (Å^2^)

**Table:**

	*U*^11^	*U*^22^	*U*^33^	*U*^12^	*U*^13^	*U*^23^
Si1	0.0274 (4)	0.0258 (3)	0.0347 (4)	0.000	0.0017 (3)	0.000
F3	0.0491 (8)	0.0408 (6)	0.0671 (8)	−0.0155 (6)	−0.0126 (6)	0.0053 (6)
O2	0.0468 (8)	0.0332 (7)	0.0463 (8)	0.0034 (6)	−0.0072 (7)	−0.0068 (6)
F004	0.0457 (8)	0.1356 (14)	0.0373 (7)	−0.0124 (8)	0.0033 (6)	−0.0186 (8)
F005	0.0401 (7)	0.0406 (7)	0.1199 (13)	0.0122 (6)	−0.0051 (8)	−0.0097 (8)
O1	0.0357 (8)	0.0381 (8)	0.0435 (8)	−0.0003 (6)	0.0016 (6)	0.0001 (6)
N1	0.0268 (8)	0.0330 (8)	0.0426 (9)	0.0010 (6)	0.0007 (7)	0.0030 (7)
C5	0.0537 (13)	0.0267 (9)	0.0406 (11)	−0.0044 (9)	0.0065 (9)	−0.0041 (7)
C1	0.0293 (10)	0.0426 (12)	0.0632 (14)	−0.0069 (9)	−0.0008 (10)	0.0046 (11)
C3	0.0383 (11)	0.0346 (10)	0.0454 (11)	0.0042 (9)	0.0074 (9)	0.0007 (8)
C011	0.0448 (12)	0.0429 (11)	0.0358 (10)	0.0049 (9)	0.0023 (9)	0.0081 (9)
C4	0.0388 (11)	0.0510 (13)	0.0390 (11)	0.0033 (9)	0.0084 (9)	0.0014 (9)
C2	0.0457 (13)	0.0414 (12)	0.0578 (13)	−0.0097 (10)	−0.0078 (11)	−0.0001 (10)
C014	0.0380 (11)	0.0505 (12)	0.0386 (11)	0.0045 (9)	−0.0064 (9)	0.0067 (9)

### Geometric parameters (Å, °)

**Table:**

Si1—F005^i^	1.6640 (13)	C1—C2	1.505 (3)
Si1—F005	1.6640 (13)	C1—H1A	0.9700
Si1—F004^i^	1.6730 (17)	C1—H1B	0.9700
Si1—F004	1.6730 (17)	C3—C4	1.503 (3)
Si1—F3	1.6836 (13)	C3—H3A	0.9700
Si1—F3^i^	1.6836 (13)	C3—H3B	0.9700
O2—C2	1.421 (3)	C011—O1^ii^	1.421 (2)
O2—C5	1.426 (2)	C011—C014	1.498 (3)
O1—C011^ii^	1.421 (2)	C011—H01A	0.9700
O1—C4	1.431 (2)	C011—H01B	0.9700
N1—C3	1.496 (2)	C4—H4A	0.9700
N1—C014	1.508 (2)	C4—H4B	0.9700
N1—C1	1.512 (2)	C2—H2A	0.9700
N1—H1	0.9100	C2—H2B	0.9700
C5—C5^ii^	1.488 (4)	C014—H01C	0.9700
C5—H5A	0.9700	C014—H01D	0.9700
C5—H5B	0.9700		
			
F005^i^—Si1—F005	92.38 (10)	N1—C1—H1B	109.7
F005^i^—Si1—F004^i^	91.18 (8)	H1A—C1—H1B	108.2
F005—Si1—F004^i^	90.36 (8)	N1—C3—C4	109.91 (16)
F005^i^—Si1—F004	90.36 (8)	N1—C3—H3A	109.7
F005—Si1—F004	91.18 (8)	C4—C3—H3A	109.7
F004^i^—Si1—F004	177.77 (13)	N1—C3—H3B	109.7
F005^i^—Si1—F3	178.75 (7)	C4—C3—H3B	109.7
F005—Si1—F3	88.72 (7)	H3A—C3—H3B	108.2
F004^i^—Si1—F3	89.40 (7)	O1^ii^—C011—C014	106.85 (16)
F004—Si1—F3	89.03 (7)	O1^ii^—C011—H01A	110.4
F005^i^—Si1—F3^i^	88.72 (7)	C014—C011—H01A	110.4
F005—Si1—F3^i^	178.75 (7)	O1^ii^—C011—H01B	110.4
F004^i^—Si1—F3^i^	89.03 (7)	C014—C011—H01B	110.4
F004—Si1—F3^i^	89.40 (7)	H01A—C011—H01B	108.6
F3—Si1—F3^i^	90.18 (10)	O1—C4—C3	111.31 (16)
C2—O2—C5	113.08 (16)	O1—C4—H4A	109.4
C011^ii^—O1—C4	114.14 (15)	C3—C4—H4A	109.4
C3—N1—C014	112.51 (15)	O1—C4—H4B	109.4
C3—N1—C1	112.39 (16)	C3—C4—H4B	109.4
C014—N1—C1	111.66 (16)	H4A—C4—H4B	108.0
C3—N1—H1	106.6	O2—C2—C1	105.91 (17)
C014—N1—H1	106.6	O2—C2—H2A	110.6
C1—N1—H1	106.6	C1—C2—H2A	110.6
O2—C5—C5^ii^	109.75 (13)	O2—C2—H2B	110.6
O2—C5—H5A	109.7	C1—C2—H2B	110.6
C5^ii^—C5—H5A	109.7	H2A—C2—H2B	108.7
O2—C5—H5B	109.7	C011—C014—N1	111.18 (16)
C5^ii^—C5—H5B	109.7	C011—C014—H01C	109.4
H5A—C5—H5B	108.2	N1—C014—H01C	109.4
C2—C1—N1	109.68 (17)	C011—C014—H01D	109.4
C2—C1—H1A	109.7	N1—C014—H01D	109.4
N1—C1—H1A	109.7	H01C—C014—H01D	108.0
C2—C1—H1B	109.7		
			
C2—O2—C5—C5^ii^	169.93 (19)	N1—C3—C4—O1	53.0 (2)
C3—N1—C1—C2	−148.05 (17)	C5—O2—C2—C1	−175.52 (16)
C014—N1—C1—C2	84.4 (2)	N1—C1—C2—O2	52.4 (2)
C014—N1—C3—C4	−150.55 (16)	O1^ii^—C011—C014—N1	60.8 (2)
C1—N1—C3—C4	82.4 (2)	C3—N1—C014—C011	76.0 (2)
C011^ii^—O1—C4—C3	88.0 (2)	C1—N1—C014—C011	−156.55 (16)

Symmetry codes: (i) −*x*+1, *y*, −*z*+1/2; (ii) −*x*+2, *y*, −*z*+3/2.

### Hydrogen-bond geometry (Å, °)

**Table:**

*D*—H···*A*	*D*—H	H···*A*	*D*···*A*	*D*—H···*A*
N1—H1···O2	0.91	2.19	2.701 (2)	115
N1—H1···O1	0.91	2.30	2.813 (2)	115
N1—H1···O1^ii^	0.91	2.37	2.826 (2)	111
C1—H1B···F004^i^	0.97	2.37	3.277 (3)	156
C011—H01B···F3^iii^	0.97	2.50	3.381 (3)	151
C2—H2A···F005^iv^	0.97	2.39	3.289 (3)	155
C014—H01C···F005	0.97	2.41	3.257 (3)	146
C3—H3A···F004^v^	0.97	2.41	3.189 (3)	137
C3—H3B···F3^iii^	0.97	2.17	3.129 (3)	169
C014—H01D···F3	0.97	2.50	3.039 (3)	115
C4—H4B···F005^v^	0.97	2.52	3.368 (3)	147

Symmetry codes: (ii) −*x*+2, *y*, −*z*+3/2; (i) −*x*+1, *y*, −*z*+1/2; (iii) −*x*+3/2, −*y*+1/2, *z*+1/2; (iv) *x*, −*y*, *z*+1/2; (v) *x*, *y*, *z*+1.
